# Differential Genome Replication of a Unique Single-Amino-Acid Mutation in the Adenovirus-4 Component of the Live Oral Adenovirus Type 4 and Type 7 Vaccine

**DOI:** 10.3390/vaccines11071144

**Published:** 2023-06-25

**Authors:** Natalie D. Collins, Shannon Beaty, Elana Wallace, Yuanzhang Li, Mark Sanborn, Yu Yang, Anima Adhikari, Paul Shabram, Kelly Warfield, Nicos Karasavvas, Robert A. Kuschner, Jun Hang

**Affiliations:** 1 Walter Reed Army Institute of Research, Silver Spring, MD 20910, USA; 2 Emergent BioSolutions, Inc., Gaithersburg, MD 20879, USA

**Keywords:** adenovirus, HAdV-4, DNA virus, vaccine, replication, mutation, pre-terminal protein

## Abstract

The FDA-approved Adenovirus Type 4 and Type 7 Vaccine, Live, Oral is highly effective and essential in preventing acute respiratory diseases (ARDs) in U.S. military recruits. Our study revealed the presence of a previously undetected mutation, not found in the wild-type human adenovirus type 4 (HAdV-4) component of the licensed vaccine, which contains an amino acid substitution (P388T) in the pre-terminal protein (pTP). This study demonstrated that replication of the T388 HAdV-4 vaccine mutant virus is favored over the wild type in WI-38 cells, the cell type utilized in vaccine manufacturing. However, results from serial human stool specimens of vaccine recipients support differential genome replication in the gastrointestinal tract (GI), demonstrated by the steady decline of the percentage of mutant T388 vaccine virus. Since vaccine efficacy depends upon GI replication and the subsequent immune response, the mutation can potentially impact vaccine efficacy.

## 1. Introduction

Human adenoviruses (HAdVs) (family *Adenoviridae*, genus *Mastadenovirus*) are non-enveloped, double-stranded DNA viruses found worldwide in humans. Greater than 80 human Ad types, belonging to species A-G, cause a diversity of illnesses, where species B, C and E can cause upper and lower respiratory tract infections and lead to severe acute respiratory disease (ARD) [1,2].

Of all human HAdVs, HAdV Type 4 (HAdV-4) and Type 7 (HAdV-7) were identified as the predominant causes of recurrent outbreaks of ARD and pneumonia in basic military recruits of all the Services (Army, Navy, Air Force and Marines) [3,4,5]. The Department of Defense (DoD), the National Institutes of Health (NIH), and Wyeth Laboratories developed a live oral vaccine comprising HAdV-4 and HAdV-7 in the 1950s and 1960s [6,7]. Following immunization of all new basic recruits, ARD rates dropped sharply and remained low [8]. The importance of universal vaccination of military recruits was affirmed after Wyeth ceased vaccine production in 1996 and the Wyeth Adenovirus Type 4 and Adenovirus Type 7 Vaccine, Live, Oral was no longer available [9]. Subsequently, HAdV ARD outbreaks recurred at US Army, Navy, Marine and Air Force Basic Training Centers, resulting in lost training time, significant morbidity and vaccine-preventable deaths [9].

**Figure 1:**
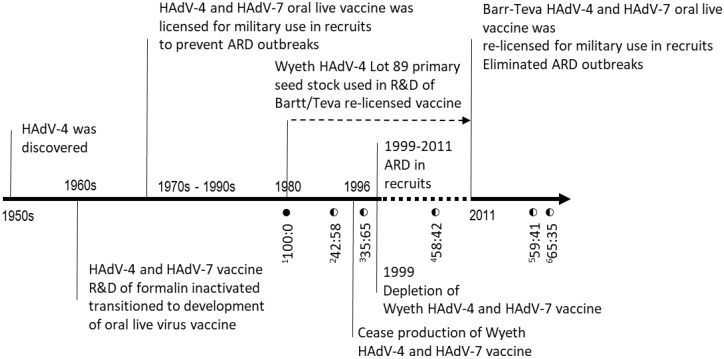
Timeline of research, development and production of human adenovirus 4 (HAd-4) vaccines. The wild-type:mutant ratio of vaccine material is presented with critical points in the timeline during Ad vaccine development and usage for the Department of Defense. Archived vaccine production seed and tablets were tested for presence of HAdV-4 vaccine strain from several time points, with the year and wild-type/mutant ratio shown. ^1^ Wyeth product seed lot 89; ^2,3^ two Wyeth HAdV-4 vaccine tablet lots (1994, 1997); ^4^ Barr Laboratories/Teva vaccine tablet used in clinical trials (2004) ^5,6^ and two recent Teva vaccine tablet lots (2017).

As a result, DoD contracted Barr (now Teva Pharmaceuticals, subsequently referred to as “Teva”) to re-establish licensure of the same vaccine. Seed lots of Wyeth vaccine viruses were obtained, and new vaccines were produced utilizing similar processes [10,11]. The Teva Adenovirus Type 4 and Type 7 Vaccine, Live, Oral received FDA licensure in 2011 and has been administered to new recruits since then, restoring effective control of HAdV-4 and HAdV-7 (Figure 1) [10,12,13].

The same vaccine strains of HAdV-4 and HAdV-7 have been used in manufacturing for over 50 years by both Wyeth and Teva. Investigating the antigenic and genetic homogeneity of the HAdV-4 and HAdV-7 vaccines has not been conducted prior to this study. Heterogeneity of other licensed live virus vaccines, including live-attenuated yellow fever virus, Polio virus and mumps virus, have been described and shown to influence phenotypic outcomes [14,15,16,17,18,19].

In this study, we discovered that the HAdV-4 component of the licensed Teva vaccine is genetically heterogenous, with a mutant virus comprising more than 35% of the viral population contained in tested vaccine materials. The mutant virus contains a single-nucleotide change (G9171T) that results in an amino acid mutation in the pre-terminal protein (pTP), encoding for a Proline (wild-type) to Threonine (mutant) substitution at position 388 (P388T). The primary known role of pTP is to act as a primer for initiation of viral DNA replication; however, much remains unknown about pTP function [20]. Mutations in some conserved regions of HAdV type 2 pTP have been shown to decrease binding to ssDNA and viral polymerase [21]. Additionally, other mutations in pTP have been shown to impair processing of mature viral particles and can lead to reduced viral infectivity of HAdV type 5 [22]. Therefore, mutations in pTP of HAdV-4 may impact the replication of HAdV and decrease virulence in wild-type strains or live, attenuated vaccine efficacy.

Interestingly, characterization of the T388 mutant virus strain identified in this study revealed that it significantly increases virus replication in the cell substrate used to propagate the vaccine virus during development and production. However, we found that the mutation negatively affects genome replication in the gastrointestinal (GI) tract of vaccine recipients (Beaty et al., 2023, manuscript submitted). The results of this study provide insight into the unexpectedly poor immunogenicity of the Live Oral Adenovirus Type 4 Vaccine (PXVX0047) developed by PaxVax (now Emergent BioSolutions, Inc., Gaithersburg, MD, USA) utilizing a vaccine tablet manufactured by Wyeth that contained only the mutant vaccine virus and highlights the contribution of pTP to replication and immunogenicity.

## 2. Methods

### 2.1. Ethics Approval

The study protocol was reviewed and granted ethical approval by the Human Subject Protection Branch (HSPB) of the Walter Reed Army Institute of Research.

### 2.2. Cell Lines and Adenovirus

Human lung adenocarcinoma A549 and human lung fibroblast WI-38 cells were obtained from ATCC and maintained in Eagle’s minimum essential media (EMEM) supplemented with 10% heat-inactivated fetal bovine serum, 1% 100× NEAA, 1% 2 mM L-glutamine, 1% penicillin/streptomycin and 0.05 mg/mL gentamicin. HAdV-4 vaccine viruses utilized in experiments were plaque purified from Adenovirus Type 4 and Type 7 Vaccine, Live, Oral tablets manufactured by Wyeth, LLC (Philadelphia, PA, USA; acquired by Pfizer in 2009) or Teva Pharmaceuticals (Parsippany, NJ, USA), as described below.

### 2.3. Virus Quantification and Plaque Purification

A549 cells are a common cell line for HAdV propagation and plaque purification, including the cytopathic effect (CPE)-based neutralization assay described previously (Beaty et al., 2023, manuscript submitted). The inner core of vaccine tablets containing HAdV-4 was extracted under sterile conditions and serially diluted 10-fold in PBS. Six-well plates containing A549 cells that were allowed to adhere for at least 18 h were infected with diluted viruses and incubated for 1 h at 37 °C in 5% CO_2_ conditions. Adherent A549 cells were then overlaid with 2% agarose prepared 1:1 with 2× EMEM supplemented with 10% FBS, 1% NEAA, 1% HEPES, 1% penicillin/streptomycin, 1% 2 mM L-glutamine and 1% 2.6 M magnesium chloride hexahydrate. Infected cells were incubated at 37 °C in 5% CO_2_ conditions for 6 days. Plaques were visualized with neutral red diluted five-fold in a 2% agarose/saline overlay and counted on Day 7 post-infection. Recovered plaques were expanded by propagating in A549 cells, and plaque purification was repeated three times. Final recovered viruses were evaluated genetically by next-generation sequencing as described below.

### 2.4. In Vitro Infection of Human Lung Fibroblast WI-38 Cells

WI-38 cells were utilized in multiple experiments to evaluate genome replication of plaque-purified wild-type and mutant HAdV-4 vaccine viruses. Our replication studies were focused on the HAdV-4 component of the vaccine due to its poor immunogenicity in human volunteers and discovery of unexpected genetic abnormalities in the vaccine virus. The HAdV-7 vaccine immunogenicity and sequence were intact as expected (Beaty et al., 2023, manuscript submitted) and, therefore, was omitted from in vitro replication studies. Prior to infection, WI-38 cells were allowed to adhere to plates at 37 °C in 5% CO_2_ for at least 18 h. Adherent cells were infected with HAdV-4 viruses in triplicate and incubated for 2 h at 37 °C in 5% CO_2_. After the inoculum was removed, cells were washed 3 times with PBS and then incubated with EMEM supplemented with 2% FBS, 1% 2 mM L-glutamine, 1% penicillin/streptomycin and 0.1% 50 mg/mL gentamicin at 37 °C in 5% CO_2_; cell supernatant was collected as described below depending on the study. For in vitro multiplication kinetic studies, WI-38 cells were infected at a multiplicity of infection (MOI) of 0.1. Cell supernatant samples were collected daily up to 10 days post-infection (dpi). Cell supernatants were assayed by qPCR to determine the viral genome-equivalent titer (GE/mL).

For the in vitro passage study, WI-38 cells were infected in triplicate with 10^5^ GE/mL of HAdVs, and cell supernatant was collected at 10 dpi, then serially passaged for a total of five passages. Viral titers in the cell supernatants from the passage study were quantified by qPCR, and nucleotide position 9171 of the viral genome was subjected to SNP genotyping to determine the frequency of the G9171T mutation. For the in vitro competition study, WI-38 cells were infected in duplicate with mixtures of wild-type P388 HAdV-4 and mutant T388 HAdV-4 viruses at ratios of 100:0, 95:5, 90:10, 75:25 and 50:50, as determined by genome-equivalent (GE) titers. Viral culture supernatants were collected at 10 dpi and subjected to SNP genotyping to determine G9171T mutation frequency.

### 2.5. Human Fecal Specimens for HAdV-4 SNP Analysis

Archived fecal samples collected during a clinical study of the Wyeth vaccine in 1998 (WRAIR # 657) from subjects vaccinated with Adenovirus Type 4 and Type 7 Vaccine, Live, Oral collected at Days 7, 14 and 21 were evaluated for the presence of wild-type and mutant vaccine viruses. A 10 to 20% of collected fecal material was previously prepared in Hanks Balanced Salt Solution (HBSS) containing 0.5% gelatin with penicillin 100 units/mL, streptomycin 100 μg/mL and 0.25 μg of fungizone. To remove the bacteria and fecal materials, the specimens were centrifuged at 2500 rpm (700× *g*) at 4 °C for 20 min, and the fecal suspension was stored at −70 °C. For genetic analysis, fecal suspensions were extracted utilizing the QIAcube and QIAamp Viral Mini RNA extraction kit (QIAGEN) and subjected to SNP genotyping.

### 2.6. Next-Generation Sequencing (NGS) and Single-Nucleotide Polymorphism (SNP) Quantitative PCR Assay

HAdV genomic DNA was extracted using the QIAamp Viral Mini RNA extraction kit (QIAGEN). The NGS DNA fragment library was prepared using the QIAseq FX DNA Library kit (QIAGEN), followed by sequencing on the MiSeq sequencing system (Illumina, San Diego, CA, USA). The HAdV-4 whole-genome sequence was assembled by mapping NGS reads using the complete genome sequence of HAdV-4 vaccine strain CL 68578 (NC_003266) as a reference. For quantitative nucleotide variation analysis by SNP-specific PCR, two primer pairs were designed with the same forward primer (5′-TGC TCA GTG ACA AAG AAG TAC ATA-3′) and with reverse primers (5′-GAG GAG GAA GTG GAG GAA CTC [C or A]-3′) differing only at the 3′ terminal nucleotides corresponding to their respective nucleotide polymorphism, i.e., G or T at nucleotide position 9171 of the HAdV-4 genome. The qPCR assay has a limit of detection (LOD) of 50 genome-equivalent (GE) copies. Non-extracted viral culture clear supernatant and extracted fecal suspensions were diluted at either a 1:5 or 1:10 ratio in water, respectively, to reduce PCR inhibitors, then combined with PowerUp SYBR Green Master Mix (Thermo Fisher) prior to being subjected to 40 cycles of quantitative PCR (qPCR) with either an ABI 7500 or ABI QuantStudio 7 Flex in duplicate. Each reaction well was quantified against an amplicon standard curve with known molecular concentration. The nucleotide percentage frequency was calculated by dividing the number of SNP-specific molecules by the total number of molecules in both SNP-respective wells (i.e., %T = 100 × T/(T + G)).

### 2.7. Statistical Analysis

Statistical analysis was completed using SAS system software. A Student’s t-test was used to compare in vitro multiplication kinetics for pTP wild-type P388 and mutant T388 viruses in WI-38 by day for each virus independently, followed by multiple comparisons through a general linear model with virus type (wild type vs. mutant), days and their interaction as predictors on the natural log value of genome equivalents (Ln GE/mL). Additionally, the over-adjusted Ln(GE/mL) average of the viruses over the 10-day period was estimated by the least-square mean. Similarly, an initial Student’s t-test, followed by a general linear model using the same parameters listed above and least-square mean, was used for the multiple comparisons of in vitro multiplication kinetics of the viruses following passaging.

A non-linear regression model by adding the square term of the percentage of input was used to evaluate the association between the percentage of the input of the pTP wild-type P388 vaccine virus and the output of the pTP wild-type P388 vaccine virus in the stool of vaccine recipients with number of days post-vaccination as predictors. Additionally, we performed multiple comparisons of the least-square mean of pTP wild-type P388 percentage by days for virus detected in stool. For the analyzed data, a value of *p* < 0.05 was considered significant.

## 3. Results

### 3.1. Discovery of Mutant Virus in Oral Live HAdV-4 Vaccine

HAdV-4 vaccine virus production primary seed stock Lot 089, manufactured in 1980 by Wyeth and also used by Teva, and several commercial vaccine tablets lots were subjected to whole-genome NGS, unpassaged, to determine genetic homogeneity (Figure 1 and Table 1). The assembled complete genome sequence for seed stock Lot 089 (1980) was identical to that of HAdV-4 vaccine strain CL 68578 (NC_003266), with no nucleotide variation detected across the entire genome. However, the HAdV-4 sequences from the tablets manufactured by Wyeth in 1994 (Lot 4948232) and 1997 (Lot 4958221) were found to contain a mixture of two nucleotides at position 9171, guanine (G) as observed in Lot 089 (1980) and thymine (T), a nucleotide substitution of G to thymine (T). G9171T is also the only detected variation across the genome shared among all Wyeth vaccine tablets sequenced. Interestingly, this mutation was also found in all the tested tablets produced by Teva for clinical trials (2004, Lot F4A023015A) and from its manufactured lots of licensed vaccine around 2017 (Lots 34600363, 34600390A) (Figure 1 and Table 1).

Results of SNP genotyping by qPCR confirmed the G/T variation frequency estimates based on sequence alignment depth of NGS data. Wyeth seed Lot 089 (1980) was determined to contain only nucleotide G9171 by SNP genotyping with no detectable T9171, while the evaluated tablets from both Wyeth and Teva contained both G9171 and T9171 (Figure 1).

To further confirm the presence of two distinct viruses in the vaccine tablets and evaluate them independently, a Teva HAdV-4 vaccine tablet (Lot 34600363, 2017) was subjected to plaque purification of viruses. Two independent HAdV-4 virus strains containing pure G9171 and T9171, respectively, were isolated and replicated stably in ten repetitive passages with genomic homogeneity confirmed by both NGS deep sequencing and SNP genotyping.

The 9171 G/T variation is within the gene for pTP, corresponding to a Proline (P)-to-Threonine (T) substitution at amino acid position 388. Only G9171 was found in Lot 089 (1980), and comparison of all HAdV-4 and other related Ad sequences available in GenBank clearly showed that G9171 is highly conserved in related Ad (Beaty et al., 2023, manuscript submitted) The T9171 variant is a novel nucleotide polymorphism among published Ad sequences and only present in the HadV-4 vaccine. Therefore, in this study, G9171 is referred to as wild-type P388, while T9171 is referred to as mutant T388.

Together, our results indicate that a G9171T mutation led to generation of a novel distinct mutant vaccine virus in the HAdV-4 vaccine. The mutant T388 vaccine virus is not detected in the early production lots of Wyeth tested in this study but was detected in all tested licensed vaccine tablets of Wyeth and Teva, at levels ranging from 35 to 65% of the viral population.

### 3.2. Mutant HAdV-4 Generates More Viral DNA than Wild-Type in WI-38 Cells

The pTP and subsequent protease-cleaved intermediate terminal protein (iTP) and mature terminal protein (TP) play multiple roles in Ad replication [20,21]. The impact on replication of the P388T mutation was evaluated in vitro in WI-38 cells, which is the same cell type utilized for vaccine virus propagation during manufacturing, although it is not the exact cell bank used during vaccine manufacturing under current good manufacturing practice (cGMP). Genome replication of the wild-type and T388 mutant viruses were compared in two experiments to examine genome replication dynamics across different timeframes. In one experiment, the virus was cultured continuously for ten days with samples collected daily, while another experiment was performed to evaluate longer-term genome replication dynamics by subjecting the viruses to five serial passages of 10 days each, with samples collected at each passage.

**Figure 2:**
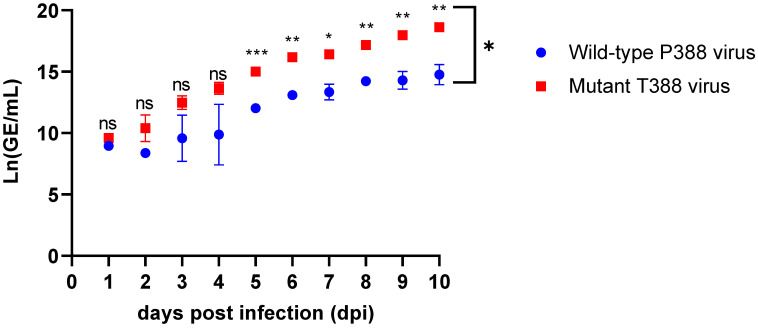
Viral genome production for wild-type P388 and mutant T388 vaccine viruses as measured by Ln(GE/mL) by day. Adherent WI-38 cells were infected at a multiplicity of infection of 0.1 and cell supernatant collected over 10 days post-infection. Multiplication kinetics of the viruses were measured by qPCR and reported at Ln(GE/mL). Data represent the mean of values obtained from three biological replicates per day. The data demonstrate that mutant T388 virus replicates to significantly higher titers as early as Day 5 post-infection. Additionally, the rate of replication of the two viruses are significantly different, the linear equation for wild-type P388 vaccine virus was Ln(GE/mL)|(p388) = 7.62 + 0.78 × Days, while mutant T388 vaccine virus was Ln(GE/mL)|(T388) = 9.16 + 1.02 × Days. *p* = 0.12 (ns), *p* = 0.033 (*); *p* = 0.002 (**) and *p* =< 0.001 (***).

DNA production of the wild-type P388 and mutant T388 vaccine viruses was evaluated in WI-38 cells individually each day for ten days and compared overall after ten days (Figure 2 and Table 2). Significant differences between wild-type P388 and mutant T388 vaccine viruses were found after Day 4, and the difference increased dramatically with each day post-infection. The results of the general linear model comparing the wild-type P388 vaccine virus to the mutant T388 vaccine virus over the 10 days demonstrated that the daily change in the rate of viral titer (Ln(GE/mL)) was significant (*p* < 0.001). Compared to the wild-type P388 vaccine virus, the overall growth rate of the mutant T338 vaccine virus is significantly higher across all time points evaluated; the average daily increase was 1.02 units of Ln(GE/mL) for the mutant T388 vaccine virus and 0.78 units of Ln(GE/mL) for the wild-type P388 vaccine virus per day (*p* = 0.003). The difference in genome produced between the two viruses calculated from the general linear model showed an increase over time by 0.24 × (number of days), ranging from 1.02 to 0.78 units of Ln(GE/mL). By Day 10 post-infection, the least-square mean of the viral titer for the wild-type P388 vaccine virus was 11.85 Ln(GE/mL) and 14.75 Ln(GE/mL) for the mutant T388 vaccine virus with a significance of *p* < 0.0001. The difference in viral genome production reached almost 1000-fold by the end of the ten-day experiment.

**Figure 3:**
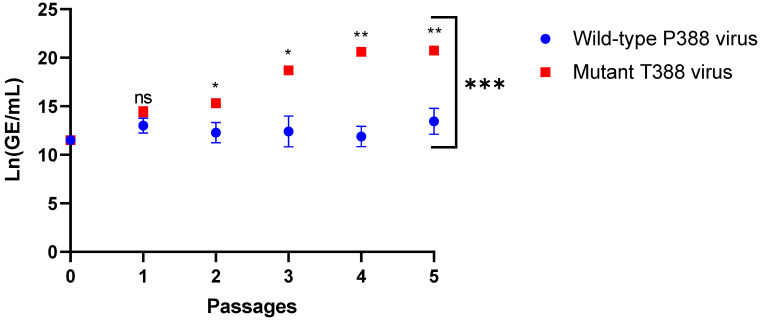
Viral genome replication for wild-type P388 and mutant T388 vaccine viruses as measured by Ln(GE/mL) over passage. Adherent WI-38 cells were infected at 10^5^ GE/mL of each virus and cell supernatant collected after 10 days post-infection and 100 µL were passaged for a total of 5 passages. Multiplication kinetics of the viruses were measured by Ln(GE/mL) after each serial passage of virus recovered from cell supernatant of WI-38-infected cells. Data represent the mean of values obtained from three biological replicates per passage. The data demonstrate that mutant T388 virus produced significantly more genomic DNA within 2 passages. Additionally, the rate of genome replication of the two viruses after serial passaging is significantly different, with linear equation for wild-type P388 vaccine virus was Ln(GE/mL)|P388 = 11.97 + 0.18 × Passage, while mutant T388 vaccine virus was Ln(GE/mL)|T388 = 12.03 + 1.94 × Passage. *p* = 0.12 (ns), *p* = 0.033 (*); *p* = 0.002 (**) and *p* =< 0.001 (***).

Similarly, genome replication after each passage for both the wild-type P388 vaccine virus and mutant T388 vaccine (Figure 3 and Table 3) and overall passage series for the wild-type P388 vaccine virus compared to the mutant T388 vaccine were determined. From Passage 2, the mutant T388 vaccine virus has significantly higher viral genomes when compared to the wild-type P388 vaccine virus. The natural logarithm of the viral genome for the wild-type P388 vaccine virus increased by 0.18 units of Ln(GE/mL) per passage with a *p* = 0.92, which was not a statistically significant increase in viral genomes after each passage. However, the natural logarithm of viral titer for the mutant T388 vaccine virus increased by 1.94 units per passage; this increase rate was found to be significant (*p* < 0.0001) and also significantly higher than that of the wild-type P388 vaccine virus (*p* < 0.0001) over the passage series.

Together, these results demonstrate that the P388T mutation confers a genome replication advantage for virus cultured in WI-38 cells. The T388 mutant vaccine virus reproduced more efficiently in WI-38 cells than the wild-type P388 vaccine virus.

### 3.3. HAdV-4 Mutant T388 Outcompeted Wild-Type P388 in WI-38 Cells

**Figure 4:**
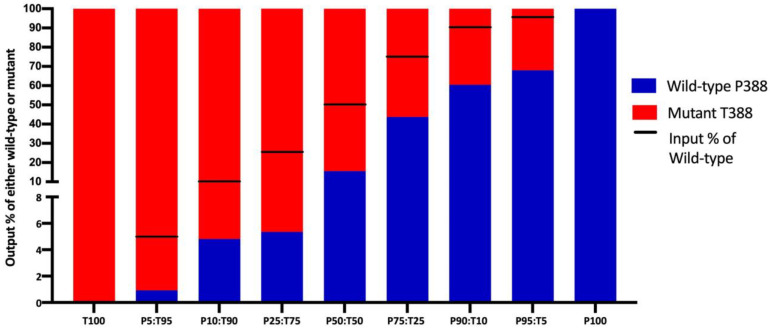
Competition of Adenovirus-4 (AdV-4) wild-type P388 and mutant T388 vaccine virus in WI-38 cells at varying P:T inputs. Adherent WI-38 cells were infected with both wild-type P388 and mutant T388 in duplicate with viral preparation of P:T mixtures to yield percentages of P95:T5, P90:T10, P75:T25 and P50:T50 mixtures and reciprocal T:P percentages as determined by qPCR; the viral preparations are termed input and the line in the graph represents the percentages of P388 in the preparation. Cell supernatants were collected 10 days post-infection and final output percentages of viruses determined by SNP genotyping. The data demonstrated that mutant T388 vaccine virus outcompetes wild-type P388 in WI-38 cells, even at lower input percentages of T388.

A competition assay of the wild-type virus and the mutant T388 virus was conducted to determine if WI-38 cells favored replication of the mutant virus in the presence of the wild-type virus. Starting with varying reciprocal percentages (ranging from GE ratio 5 to 95% of input virus), the final percentage (output virus) of the wild-type P388 to the mutant T388 vaccine virus following 10-day infection in WI-38 cells was determined by SNP qPCR (Figure 4).

When starting with 5% of mutant T388 virus in the mixture (GE ratio of T5:P95), the mutant T388 virus increased to 32%, while the wild type decreased from 95% to 68% (output GE ratio of T32:P68). Similarly, the input mixtures of T10:P90 and T25:P75 resulted in output ratios of T40:P60 and T56:P44, respectively. When starting with an equal mix of the wild-type and the mutant T388 viruses (T50:P50), mutant T388 clearly outcompeted, ending with a ratio of T84:P16. Interestingly, when starting with an input GE ratio of only 5% wild-type P388 virus, limited replication was observed and the proportion dropped to less than 1% wild-type P388 recovered.

### 3.4. Mutant HAdV-4 Was Replication Deficient in Human Gastrointestinal Tract

**Figure 5:**
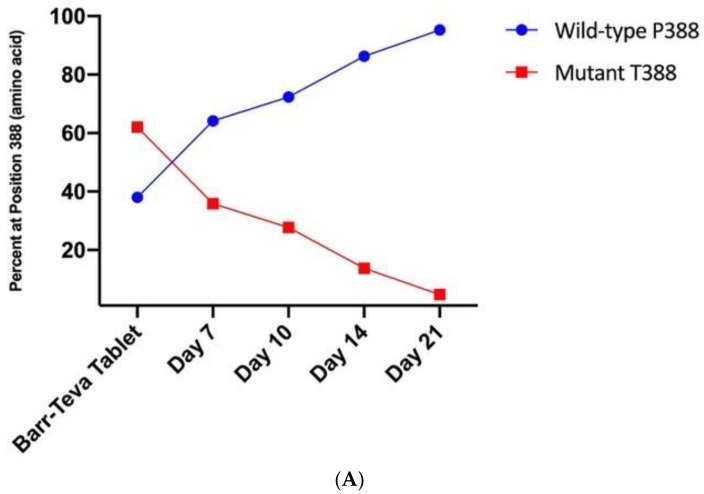

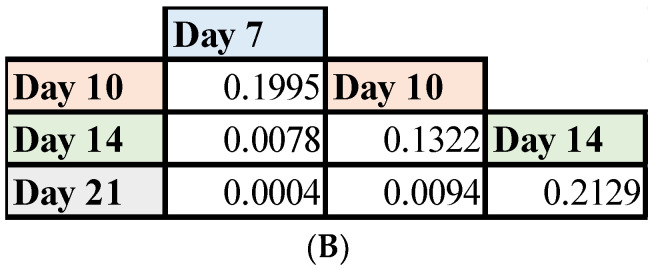
Percentage of wild-type P388 and mutant T388 vaccine virus recovered from archived stool of live oral adenovirus vaccine recipients. Vaccine recipients were co-administered live oral Adenovirus Type 4 and Type 7 Vaccine and stool samples were collected on Days 7, 10, 14 and 21. The proportion of wild-type P388 and mutant T388 vaccine viruses was determined by SNP PCR assay. The proportion of wild-type P388 to mutant T388 vaccine virus in the AdV-4 component was P64:T36; the percentage of wild-type P388 measured in clinical specimens at various time points post-vaccination is shown in panel (**A**). Panel (**B**) shows *p*-values for the least-square average of the wild-type percentage by day of wild-type P388 vaccine virus. Wild-type P388 vaccine virus steadily increases in stool of vaccine recipients, with Day 7 significantly differing from Days 14 and 21; within 21 days post-vaccination, wild-type P388 represents the predominate genotype in the stool of vaccine recipients.

The Wyeth and Teva Ad live vaccines are administered orally; the virus replicates in the GI tract of vaccine recipients and is shed in stools for as long as 28 days. Replication in the GI tract is a critical step for a protective immune response. With the discovery that the Wyeth and Teva HAdV-4 vaccines comprise a mixture of wild-type and mutant strains, we investigated whether the differential replication that we demonstrated in vitro with plaque-purified viruses also occurs in the human GI tract. Archived stool specimens from volunteers who received the Wyeth HAdV-4 and HAdV-7 vaccine were subjected to SNP genotyping (Figure 5). The percentage of wild-type P388 to mutant T388 in the tablets utilized during the study was 38% to 62%, i.e., P38:T62. The fecal specimens were collected on Days 0 (prior to vaccination), 3, 7, 14, 21 and 28. No HAdV-4 was detected in any specimens collected on Days 0, 3 and 28. The average ratios in stools for Days 7, 14 and 21 were P64:T36, P86:T14 and P95:T5, respectively. The results of the least-mean pairing test showed the percent of the wild-type P388 vaccine virus in stools on Days 14 (*p* = 0.0078) and 21 (*p* = 0.0004) post-vaccination was significantly different when compared to the initial percentage of the wild type in the HAdV-4 tablet administered to vaccines (Figure 5B). During this time period, the difference in average percent of wild-type increased by 2.36% (*p* = 0.0001) per day.

## 4. Discussion

Licensed Adenovirus Type 4 and Type 7 Vaccine, Live, Oral have been in use by DoD for over 50 years, during which time two different manufacturers, Wyeth and Teva, produced essentially the same vaccine [6,7,10,11,13]. This study showed, for the first time, that HAdV-4 vaccine tablets produced by both manufacturers since the 1990s contain a mixture of wild-type P388 and mutant T388 vaccine strains. Amino acid P388 in the pTP is highly conserved among both wild-type human and animal adenoviruses, with the P388T mutation being unique to the HadV-4 component of the Adenovirus Type 4 and Type 7 Vaccine, Live, Oral [23,24,25] (Beaty et al., 2023, manuscript submitted). Few studies have investigated the genetic diversity of double-stranded DNA viruses, like HadV-4, mainly because DNA viruses possess high-fidelity polymerases and mechanisms for DNA repair, which result in a low rate of replication error. However, a study of Ad5 by Risso-Ballester et al. showed evidence of sequence heterogeneity and mutation hotspots, but none at P388 [26]. This suggests adenoviruses undergo spontaneous mutations given the right conditions and may present a mechanism for the introduction of the P388T mutation. Mutations of vaccine viruses have been identified in live RNA virus vaccines. Specifically, studies on the live-attenuated mumps vaccine made from strain Urabe AM9 showed that growth conditions had encouraged the development of virus mutations, some of which were associated with altered phenotypic effects [16,17]. In this study, NGS, SNP genotyping by qPCR and virological assays of the HAdV-4 vaccine wild-type virus and the novel T388 mutant virus revealed the functional importance of the P388 residue of pTP and the emergence and accumulation of the T388 mutant during vaccine production.

A seed-lot system was used during vaccine virus propagation in which WI-38 cells were infected with stock vaccine virus and cell supernatant was collected, then passaged multiple times to generate aliquots of working seed virus stocks, which then undergo additional passages to produce vaccine drug substance lots for tablet production [27,28]. Experimental conditions used in this study attempted to replicate commercial vaccine manufacturing propagation conditions, albeit at a much smaller scale, with wild-type P388 and mutant T388 HAdV-4 vaccine viruses being subjected to limited passaging in WI-38 cells. Evaluation of the viral genome replication in vitro revealed that the mutant T388 HAdV-4 vaccine virus replicates to higher titers (GE/mL) in WI-38 cells. Serial passaging of the two strains further demonstrated a significantly greater viral genome for the mutant T388 HAdV-4 vaccine virus. In vitro competition assays clearly showed that the T388 mutant outcompetes the wild type and overwhelmingly predominates the population within the span of only 10 passages in WI-38 cells. We speculate that the P388T mutation may have originated during the vaccine manufacturing process as the virus was propagated in WI-38 cells [28]. To our knowledge, the primary seed stock Lot 089 HadV-4 vaccine virus (1980) was the only viral stock shared between the two manufacturers. Thus, we hypothesize that the same spontaneous mutation was generated due to adaption to growth in WI-38 cells; thus, generation of the mutation is a cell culture adaption for growth in WI-38 cells such that both manufacturers yield similar mutations when propagating vaccine work virus stocks or drug substance material. Alternatively, Lot 089 may have contained the T388 mutant at titers too low to detect by either NGS or SNP genotyping methods used in this study. Further analysis of all archived manufacture data and retained production samples could shed light on the origin of the mutant T388 vaccine virus.

Interestingly, although the mutant T388 vaccine virus outcompeted the wild type in vitro, we found that its replication in the GI tract of human vaccines was diminished relative to the wild-type P388 vaccine virus. The drastic increase in the P388-to-T388 ratio in fecal specimens collected post-vaccination suggests that the wild-type virus established a more robust infection and prolonged replication in the GI tract of vaccines, outcompeted the T388 mutant virus, and eventually became the predominant strain. The differential genome replication observed in vitro and the concomitant decline in the percentage of T388 in the stool of vaccine recipients could indicate decreased production of infectious virus particles; further investigation is needed to fully explore this hypothesis.

The oral HAdV vaccines produce transient asymptomatic infection of the GI tract, thereby inducing a type-specific protective immune response that is highly correlated with the presence of serum broadly neutralizing antibody titers [12,27,28,29]. Replication of the T388 mutant vaccine virus in the GI tract of the vaccine recipient has the potential for reducing vaccine effectiveness. In fact, this outcome was observed in a clinical trial of a plasmid-derived HAdV-4 vaccine PXVX0047 developed by PaxVax (now Emergent BioSolutions Inc), which contained only mutant T388, and resulted in low HAdV-4 seroconversion rates (Clinical Trial NCT03160339), (Beaty et al., 2023, manuscript submitted). We hypothesize that the use of pure T388 vaccine virus in the PXVX0047 vaccine may have contributed to the low seroconversion rates. Continued changes in the proportion of mutant virus in the current licensed vaccine could adversely affect vaccine efficacy and this warrants further study. Therefore, closely monitoring wild-type and mutant proportions during vaccine production and each lot of vaccine tablets would ensure the biological integrity of the product.

Our discovery of the significant functional impact of the P388T mutation raises questions about its mechanism of action and underscores the need for further investigation of the role and structure of the pTP. Furthermore, this study highlights the importance of verifying findings from in vitro studies in relevant tissues and organs of human vaccine recipients. More systematic comparative analyses are needed to shed light on how a single-amino-acid substitution at this residue results in a drastic phenotypical change and what factors modulate the differential replication in an in vivo versus in vivo environment.

## Figures and Tables

**Table 1:** Genetic diversity at nucleotide 9171 of various adenovirus type 4 vaccine viruses and tablets.

Material ID	Material Type	Manufacturer	Nucleotide Percentages (%) at Position 9171	
			G9171	T9171
HAdV-4 CL68578	Vaccine reference strain	Wyeth	100	0
Lot 089	Seed stock	Wyeth	100	0
Lot 4948232	Vaccine tablet	Wyeth	42	58
Lot 4958221	Vaccine tablet	Wyeth	35	65
Lot F4A023015A	Vaccine tablet	Teva	58	42
Lot 34600363	Vaccine tablet	Teva	65	35
Lot 34600390A	Vaccine tablet	Teva	59	41

**Table 2:** The mean Ln(GE/mL) difference of in vitro viral genome production in WI-38 cells by day between T388 and P388 using the Student’s *t*-test.

Days	Mean Ln(GE/mL) P388	Mean Ln(GE/mL) T388	Mean Ln(GE/mL) Difference	Mean Difference StdErr *	*p*-Value *
1	8.96	9.9	0.63	0.18	0.06
2	8.37	10.38	2.02	0.63	0.09
3	9.57	12.48	2.91	1.13	0.11
4	9.87	13.63	3.76	1.77	0.27
5	12.02	15.01	2.99	0.15	<0.0001
6	13.09	16.18	3.09	0.26	0.001
7	13.34	16.42	3.08	0.38	0.01
8	14.22	17.17	2.95	0.21	0.0005
9	14.29	17.98	3.70	0.46	0.004
10	14.75	18.63	3.88	0.49	0.01

* Mean difference of standard error (StdErr) and *p*-value are from Satterthwaite test for unequal variances.

**Table 3:** The mean Ln(GE/mL) difference of in vitro viral genome production following passaging in WI-38 cells between T388 and P388 using the Student’s t-test.

Passage	Mean Ln(GE/mL) P388	Mean Ln(GE/mL) T388	Mean Ln(GE/mL) Difference	Mean Difference Standard Error	*p* * Value
0	11.51	11.51	0.00	0.00	1.0
1	13.02	14.44	1.42	0.53	0.07
2	12.27	15.31	3.04	0.61	0.04
3	12.40	18.70	6.31	0.92	0.02
4	11.88	20.61	8.73	0.63	0.003
5	13.44	20.73	7.29	0.77	0.01

* *p*-value is from Satterthwaite test for unequal variances.

## Data Availability

The HAdV-4 vaccine virus seed stock and tablets listed in Table 1 were subjected to whole-genome sequencing using Illumina MiSeq system. All NGS raw read data were deposited in the SRA database under BioProject PRJNA961906 (https://www.ncbi.nlm.nih.gov/sra/PRJNA961906).
